# Ferulic Acid Ameliorates Lipopolysaccharide-Induced Barrier Dysfunction *via* MicroRNA-200c-3p-Mediated Activation of PI3K/AKT Pathway in Caco-2 Cells

**DOI:** 10.3389/fphar.2020.00376

**Published:** 2020-04-03

**Authors:** Shasha He, Yuhong Guo, Jingxia Zhao, Xiaolong Xu, Ning Wang, Qingquan Liu

**Affiliations:** ^1^ Beijing Hospital of Traditional Chinese Medicine, Capital Medical University, Beijing, China; ^2^ Beijing Institute of Traditional Chinese Medicine, Beijing, China; ^3^ Beijing Key Laboratory of Basic Research with Traditional Chinese Medicine on Infectious Diseases, Beijing, China

**Keywords:** ferulic acid, intestinal epithelial barrier, Caco-2 cells, miR-200c-3p, PTEN/PI3K/Akt pathway

## Abstract

Intestinal barrier dysfunction is an important clinical problem in various acute and chronic pathological conditions. Ferulic acid (FA) can attenuate the intestinal epithelial barrier dysfunction, however, the underlying mechanism remains unclear. The present study aimed to uncover the protective effect of FA on intestinal epithelial barrier dysfunction in a Caco-2 cell model of lipopolysaccharide (LPS) stimulation and the underlying mechanism. Caco-2 cells were pretreated with FA and then exposed to LPS stimulation. The barrier function of Caco-2 cells was evaluated by measuring trans-epithelial resistance (TER) and 4-kDa fluorescein isothiocyanate (FITC)-dextran (FD4) flux, and analyzing the tight junction protein expression and structure. The results showed that decreased TER and increased FITC-FD4 flux were observed in Caco-2 cells stimulated with LPS, but these effects were attenuated by FA pretreatment. FA pretreatment inhibited LPS-induced decrease in occludin and ZO-1 mRNA and protein expression. LPS stimulation decreased miR-200c-3p expression, whereas this decrease was inhibited by FA pretreatment. Furthermore, overexpression of miR-200c-3p strengthened the protective effects of FA on LPS-induced Caco-2 cell barrier dysfunction by decreasing epithelial permeability, increasing occludin and ZO-1 protein expression, and maintaining of ZO-1 protein distribution, while suppression of miR-200c-3p reversed the protective effects of FA. LPS treatment increased the expression of PTEN protein and decreased expression of phosphorylated PI3K and AKT proteins. However, pretreatment of FA inhibited expression of PTEN protein and promoted activation of PI3K/AKT signaling pathway in the LPS-treated Caco-2 cells, and this regulatory effect of FA on the PTEN/PI3K/AKT signaling pathway was strengthened or weakened by miR-200c-3p overexpression or suppression, respectively. Our findings suggested that in Caco-2 cells, FA promotes activation of PI3K/AKT pathway by miR-200c-3p-mediated suppression of the negative mediator PTEN, which, in turn, maintains TJ function and thus ameliorates LPS-induced intestinal epithelial barrier dysfunction.

## Introduction

The intestinal tract is lined with a single layer of epithelial cells that acts as a selective barrier, allowing absorption of nutrients, electrolytes, and water while preventing the transfer of intestinal pathogens, antigens and toxins from the luminal environment to blood circulation and mesenteric lymph (Odenwald and Turner, 2017). Dysfunction of intestinal epithelial barrier may lead to increased permeability of intestinal mucosa, subsequent translocation of intestinal pathogenic bacteria or toxins, which in turn aggravates the damage of intestinal barrier integrity, resulting in local intestinal or systemic disease such as inflammatory bowel diseases, multiple organ dysfunction syndromes and sepsis (Michielan and D’Inca, 2015; Zhou and Verne, 2018). Therefore, maintenance of the intestinal epithelial barrier function is very important in the clinical treatment of various acute and chronic diseases.

Tight junctions (TJs), located at the apical side of the lateral membranes of intestinal epithelial cells, are the primary factors in determining of paracellular permeability (Buckley and Turner, 2018). TJs are composed of a variety of proteins, in which the transmembrane protein occludin and the cytoplasmic protein zonula occludens-1 (ZO-1) are key proteins that maintain TJs structure and intestinal epithelial barrier function (Costantini et al., 2009). TJs can be compromised by luminal noxious antigens, one of which is lipopolysaccharides (LPS), the main component of the outer membrane of Gram-negative bacteria. Growing investigations indicate that LPS triggers an inflammatory signaling cascade to reduce tight junction protein expression, leading to increased intestinal permeability and disrupting intestinal epithelial barrier function (He et al., 2019; Tunisi et al., 2019). Clinical study also demonstrated that circulating blood LPS levels are elevated in Crohn’s disease and sepsis patients, and contribute to the pathogenesis of intestinal and systemic inflammatory response (Guo et al., 2015).

Ferulic acid (4-hydroxy-3-methoxycinnamic acid, FA) is a natural phytochemical widely found in vegetables and fruits, and also is the main active ingredient in many traditional Chinese medicines, such as *Angelica sinensis, Ligusticum chuanxiong,* and *Cimicifuga foetida* (Mancuso and Santangelo, 2014). It is a derivative of curcumin and has the necessary pharmacokinetic properties to be retained for several hours in general circulation (Ghosh et al., 2017). A great amount of evidence indicates that FA has strong antioxidant, anti-inflammatory and anti-apoptotic pharmacological properties (Shanthakumar et al., 2012; Mhillaj et al., 2018). It is also shown that FA has protective effects against Alzheimer’s disease, cardiovascular diseases, and sepsis (Bacanli et al., 2014; Sgarbossa et al., 2015). However, the underlying mechanisms by which FA attenuates LPS-induced intestinal epithelial barrier dysfunction have not been clarified.

MicroRNAs (miRNAs) are a series of small non-coding RNA molecules (containing 20–25 nucleotides) found in eukaryotes that participate in RNA silencing, post-transcriptional, and translational regulation of gene expression (Ambros, 2004). Recent evidence has shown that miRNAs play a vital role in controlling intestinal epithelial barrier function, in part by regulating the expression of tight junction proteins (Martinez et al., 2017). MiR-200c is a member of the miR-200 family, in which miR-200b and miR-429 have been shown to be involved in the regulation of intestinal epithelial barrier function (Yu et al., 2016; Shen et al., 2017). Many reports revealed that miR-200c plays a key role in the epithelial-mesenchymal transition, apoptosis, proliferation, and metastasis of various cancer cells (Mutlu et al., 2016; Zhou et al., 2018). However, the role of miR-200c in intestinal epithelial barrier function remains unclear.

PTEN, a dual-specificity phosphatase, has been demonstrated to be a possible target of miR-200c (Liao et al., 2013). It is well known that PTEN competes with the PI3K/AKT activity. Activated AKT pathway regulates multiple biological processes such as cell survival, autophagy, and apoptosis (Liao et al., 2013). Our previous studies showed that FA has a protective effect on intestinal epithelial barrier function in IEC-6 cells suffering from heat stress, which is associated with activation of the antioxidant PI3K/AKT/Nrf2/HO-1 signaling pathway (He et al., 2016; He et al., 2018). In the present study, we investigated the underlying mechanism by which FA regulates intestinal epithelial barrier function *via* miR-200c-3p/PTEN/PI3K/AKT pathway in an LPS-induced Caco-2 monolayer barrier dysfunction model.

## Materials and Methods

### Cell Culture and Treatment

The human colon cancer cell lines (Caco-2) were obtained from the Cell Resource Center, Peking Union Medical College (Beijing, China). Cells were maintained in Rosewell Park Memorial Institute (RPMI) 1640 medium (Gibco, NY, USA) containing 20% fetal bovine serum (FBS, Gibco), 100 U/ml penicillin and 0.1 mg/ml streptomycin in a humidified (37°C and 5% CO2) incubator. For LPS treatment, Caco-2 cells were incubated in RPMI 1640 and different concentrations of LPS (Sigma-Aldrich, St. Louis, USA) for 24 h.

FA (99% purity, CAS 110773-201614), purchased from the National Institutes for Food and Drug Control (Beijing, China), was dissolved in RPMI 1640 to 10 mM as a stock solution. The experiment concentrations of FA were 0, 25, 50, and 100 μM. Cells were pretreatment with FA for 2 h before LPS treatment.

### Intestinal Epithelial Barrier Function Determination

Transepithelial electrical resistance (TER) of filter-grown Caco-2 monolayers was measured using a Millicell-ERS system (Millipore, Bedford, USA). Cells were seeded on transwell inserts (Corning, Cambridge, USA) with polyethylene terephthalate membrane (0.33 cm^2^, 0.4 μm pore size) at a density of 1.0 ×10^6^ cells/well and monitored daily for about 21 days. When cells reached confluence and completely differentiated, different concentration FA (25, 50, or 100 μM) were added to the apical of the filter for 2 h and then treated with or without LPS. Changes in TER under experimental conditions were expressed as a percentage of the corresponding basal values. Determinations were repeatedly operated on three different sites transwell insert, respectively.

The permeability of the epithelial across Caco-2 cell monolayers was quantified by measuring the flux of the FITC-labeled dextran of molecular mass 4 kDa (FD4; Sigma-Aldrich, St. Louis, USA). In brief, 1 mg/ml of FD4 was added to the apical chamber and the medium was collected from the basolateral chamber after 2 h’s incubation. Fluorescence intensity of FITC was quantified using a fluorescence plate reader (BioTek, Winooski, USA) with excitation at 492 nm and emission at 520 nm.

### Cell Viability Assay

Cell viability was determined using Cell Counting Kit-8 (CCK-8; Dojindo, Kumamoto, Japan) assay according to the manufacturer’s instructions. In brief, Caco-2 cells (1 × 10^4^ cells) were plated on a 96-well plate and incubated at 37°C with 5% CO_2_ for 24 h. Cells were then washed twice with PBS and incubated with LPS (0.001, 0.01, 0.1, 1, 10, or 100 μg/ml) for 24 h or FA (1, 5, 10, 20, 50, 100, 200, or 500 μM) for 48 h. The cells were then treated with 10 µl/well of CCK-8, and incubated at 37°C for 2 h. Absorbance was detected at 450 nm using a microplate reader (PerkinElmer, Inc., Waltham, USA).

### Transmission Electron Microscopy (TEM)

Fully confluent cultured Caco-2 cells were pretreated with 100 μM FA for 2 h and then subjected with LPS for 24 h. Cells were scraped off and centrifuged at 1500 rpm for 10 min. The samples fixed in overnight with 4% glutaraldehyde and then post-fixed in cold 1% osmium tetroxide for 1 h, followed by three cacodylate buffer washes. After dehydration in graded ethanol solutions, cells were embedded in Epon-Araldite (EPON 812, Emicron, Shanghai, China). Ultra-thin sections were stained with saturated uranyl acetate in 50% ethanol and lead citrate and examined with transmission electron microscopy (H7650, Hitachi, Ltd., Tokyo, Japan).

### Analysis of miRNA Expression Profile

Total RNA from Caco-2 cells was extracted using Trizol reagent (Life Technologies, Carlsbad, USA) according to the manufacturer’s protocol. The quality and quantity of the RNA samples were assessed on a Bioanalyzer 2100 system (Agilent Technologies, Santa Clara, USA) using an RNA 6000 Nano kit (Agilent Technologies). Small RNA libraries were generated according to the protocol of Illumina TruSeq™ Small RNA Sample Preparation kit (Agilent Technologies). Affymetrix GeneChip miRNA array analysis was conducted to detect the expression pattern of miRNAs, which were provided by Shanghai Biotechnology Corporation (Shanghai, China). Bioinformatic analyses and visualization of microarray data were performed with MultiExperiment Viewer (MEV) software v.4.6 (TIGR, La Jolla, USA).

### Cell Transfection

Lentivirus-miR-200c-3p (Lv-miR-200c-3p) or lentivirus-miR-200c-3p sponge (Lv-miR-200c-3p spong) were transfected into Caco-2 cells in order to increase or inhibit miR-200c-3p expression, respectively, and lentivirus-negative control (Lv-NC) was used as negative control. The Lv-miR-200c-3p, Lv-miR-200c-3p spong and Lv-NC were designed and synthesized by Hanheng Biotechnology Corporation (Shanghai, China). Concisely, 293T cells were co-transfected with viral packaging vector pMD2.G and psPAX2 (Addgene, Cambridge MA), along with a lentiviral construct expressing a specific miR-200c-3p, miR-200c-3p spong or the empty vector, using LipoFitter™ transfection reagent (Hanheng Biotechnology Corporation) according to the manufacturer’s instructions. The procedure of miR-491-3p synthesis was the same as that of miR-200c-3p. The transfection medium was replaced after 6 h with fresh Dulbecco’s Modified Eagle Medium (Gibco, NY, USA) containing 10% FBS, 100 U/ml penicillin and 0.1 mg/ml streptomycin. At 48 h after incubation, the supernatant of lentivirus was ultracentrifuged, concentrated and used to infect cells, as previously described (Wu et al., 2019). In brief, Caco-2 cells (1 × 10^6^ cells/well) seeded on a 6-well plate were transfected with Lv-miR-200c-3p, Lv-miR-200c-3p spong or Lv-NC at a multiplicity of infection (MOI) of 15, or transfected with Lv- miR-491-3p, Lv- miR-491-3p spong or Lv-NC at a MOI of 20, and incubated at 37°C with 5% CO2 for 48 h. Transfection efficiency was confirmed by quantitative reverse transcription PCR. Then, the cells were treated according to different test requirements.

### Quantitative Real-Time PCR

Total RNA was extracted using TRIzol Reagent (Life Technologies) according to the manufacturer’s protocol. The cDNA was reverse transcribed using the revert aid first-strand cDNA Synthesis kit (Thermo Fisher Scientific). Real-time PCR was performed using SYBR Green qPCR Master Mix (Applied Biosystems). The Occludin, ZO-1 and β-actin primer sequences were synthesized by Sangon Biotech (Shanghai, China). The primers sets were as follows: Occludin, 5′-GCAAAGTGAATGACAAGCGG-3′ (F) and 5′-CACAGGCGAAGTTAATGGAAG-3′ (R); ZO-1, 5′-CGAAGGAGTTGAGCAGGAAA-3′ (F) and 5′-ACAGGCTTCAGGAACTTGAG-3′ (R); β-actin, 5′-TGCAGAAAGAGATCACCGC-3′ (F) and 5′-CCGATCCACACCGAGTATTTG-3′ (R). To generate cDNA of miR-200c-3p, total RNA was reverse transcribed using a miRcute miRNA cDNA Kit (TIANGEN BIOTECH, Beijing, China) according to the manufacturer’s protocol. Real-time PCR was performed using miRcute miRNA (TIANGEN BIOTECH) according to the manufacturer’s protocol. The primers of miR-200c-3p and U6 were purchased from Guangzhou Ruibo Biotechnology Co. LTD. All real-time qPCR analyses were conducted using the 7500 Real-Time PCR system (Applied Biosystems). Relative expression of mRNA and miR-200c-3p were normalized using the endogenous controls β-actin and U6, respectively. The amplification protocols were as follows: 95°C for 10 min, 40 cycles of 95°C for 15 s, 60°C for 34 s, and 72°C for 34 s. Relative expression fold changes were calculated using the 2^−ΔΔCT^ method.

### Western Blotting

Total proteins from Caco-2 cells were extracted on ice using RIPA buffer (Beyotime Company, Beijing, China) supplemented with protease inhibitors (Roche, Basel, Switzerland). The concentration of total proteins was measured using the BCA protein assay kit (Beyotime, Jiangsu, China). Then, specified amounts of protein samples were separated by SDS-PAGE and electrotransferred onto nitrocellulose membranes (Pierce, Waltham, MA). After blocking with Odyssey blocking buffer (LI-COR Biosciences, NE, USA) for 2 h, the membranes were incubated with primary antibodies rabbit anti-occludin (1:100, 71–1500, Invitrogen, Carlsbad, USA), rabbit anti-ZO-1 (1:500, 21773-1-AP, Proteintech, Wuhan, China), rabbit anti-PTEN (1:1000, 9559, Cell Signaling Technology, Danvers, MA), rabbit anti-PI3K (1:1000, ab191606, Abcam, Cambridge, United Kingdom), rabbit anti-phospho-PI3K (1:1000, ab182651, Abcam), rabbit anti-Akt (1:1000, 4685, Cell Signaling Technology), rabbit anti-phospho-Akt (1:1000, 4060, Cell Signaling Technology), and rabbit anti-β-actin (1:2000, 4970, Cell Signaling Technology) overnight at 4°C. The membranes were washed three times and incubated with horseradish peroxidase (HRP)-conjugated goat-anti-rabbit IgG secondary antibody (1:5000, A8275, Sigma-Aldrich) for 30 min at room temperature. Then, the protein bands were visualized using enhanced electrogenerated chemiluminescence western blotting detection reagents (Pierce Corporation, Rockford, IL). The gray values of the target band of proteins were quantified using Image J (National Institutes of Health, Bethesda, MD).

### Immunofluorescence Staining

Caco-2 cells were cultured on coverslips to fully confluence and subjected to various experimental conditions. After treatment, cells were washed with PBS three times and fixed with 4% paraformaldehyde for 10 minutes at room temperature. Then cells were permeated with 0.1% Triton X-100 for 5 min, followed by blocking with 3% BSA for 1 h at room temperature. Caco-2 cells were incubated with rabbit anti-ZO-1 antibody (1:50, 21773-1-AP, Proteintech) at 4°C overnight. Coverslips were washed and incubated with a secondary antibody Alexa Fluor 488 goat anti-rabbit IgG (1:400, A-11008, Life Technologies, Carlsbad, USA) for 40 min at room temperature in the dark. Then, Cells were counterstained with 4′,6-diamidino-2-phenylindole (DAPI) (Beyotime, Jiangsu, China) for 5 min at room temperature and visualized with a fluorescence microscope (Olympus IX71, Tokyo, Japan).

### Statistical Analysis

All data were represented as means ± standard deviation (SD). Statistical analysis was performed using the GraphPad Prism 7 program (GraphPad, La Jolla, USA). For data from assay evaluating the effect of LPS on TER values and FD4 flux, the unpaired *t* test was used. One-way analysis of variance (ANOVA) was performed to compare the statistical differences of data among three or more groups. A *P*-value of <0.05 was considered statistically significant. All experiments in this study were repeated at least three times.

## Results

### LPS Induced Intestinal Epithelial Barrier Dysfunction in Caco-2 Cells

**Figure 1:**
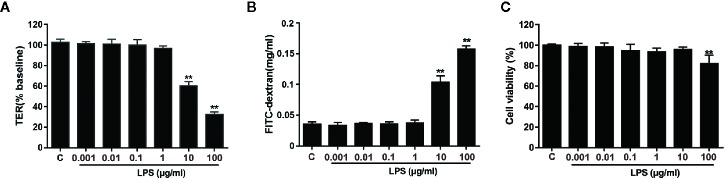
Effects of LPS on TER and FD4 permeability in Caco-2 monolayers. Caco-2 cells grown on transwell inserts were treated with various concentrations (0.001 − 100 μg/ml) of LPS for 24 h. TER **(A)** and FD4 flux **(B)** across cell monolayers were measured. **(C)** The CCK-8 assay was used to detect the viability of cells infected with LPS (0.001, 0.01, 0.1, 1, 10, or 100 μg/ml). Data are presented as means ± SD from three independent experiments. Statistical analyzed using the unpaired *t* test. *P < 0.05, **P < 0.01.

Firstly, the effects of different concentrations of LPS (0.001 − 100 μg/ml) on epithelial paracellular permeability and cell viability were determined by measuring TER values and FD4 flux in Caco-2 cell monolayers. **Figure 1A** displayed that LPS at a concentration of above 10 μg/ml significantly decreased the TER value (*P* < 0.01), whereas LPS at a concentration of below 10 μg/ml had no significant effect on the TER. **Figure 1B** presented that 10 μg/ml or 100 μg/ml LPS significantly increased the FD4 flux from Caco-2 monolayer apical to the basolateral chamber (*P* < 0.01). **Figure 1C** showed that 10 μg/ml LPS had no effect on the cell viability, while 100 μg/ml LPS decreased the cell viability. Consequently, we applied 10 μg/ml LPS concentration to induce intestinal epithelial barrier dysfunction for subsequent experiments.

### FA Alleviated LPS-Induced Intestinal Epithelial Barrier Dysfunction

**Figure 2:**
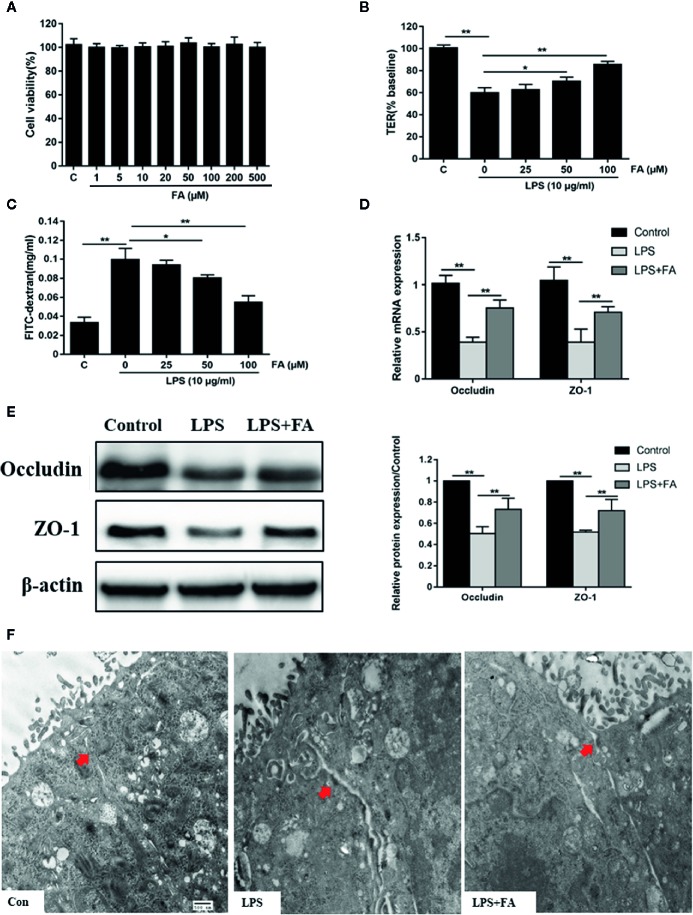
The protective effects of FA on LPS-induced intestinal epithelial barrier dysfunction. **(A)** Caco-2 cells were incubated with FA (0 - 500 μM) for 48 h and then CCK-8 assay was used to detect cell viability. **(B, C)** Cells were pretreated with FA (25, 50, 100 μM) for 2 h and then stimulated with LPS for 24 h. TER and FD4 flux were measured to evaluate the paracellular permeability. **(D, E)** Caco-2 cells were pretreated with 100 μM FA for 2 h and then stimulated with LPS for 24 h. The mRNA and protein expression levels of occludin and ZO-1 were determined by qRT-PCR and Western blot analysis. **(F)** Ultrastructure of TJs in Caco-2 monolayers cell was observed with a transmission electron microscope (black arrow indicated, Scale bar = 500 nm). Data were presented as means ± SD from three independent experiments and differences between means were compared using one-way ANOVA with Tukey’s multiple comparisons test. *P < 0.05, **P < 0.01.

Effects of FA on LPS-induced intestinal epithelial barrier dysfunction were subsequently explored. To determine whether FA had toxic effects, Caco-2 cells were incubated with 0−500 μM FA for 48 h and cell viability was assessed. In **Figure 2A**, there were no statistical differences in cell viability, regardless of dose of FA, indicating 0−500 μM FA showed no cytotoxic effects on Caco-2 cells. Then, Caco-2 cells were pretreated with FA (25, 50, and 100 μM) for 2 h, followed by LPS stimulation. **Figure 2B** displayed that compared with LPS-treated cells, cells pretreated with 50 and 100 μM FA had higher TER values (*P* < 0.05 and *P* < 0.01). Similarly, the LPS-induced increase in FD4 flux across the Caco-2 monolayer was significantly attenuated by 50 and 100 μM FA (*P* < 0.05 and *P* < 0.01, **Figure 2C**).

Compared with control cells, decreased mRNA (*P* < 0.01, **Figure 2D**) and protein (*P* < 0.01, **Figure 2E**) expression levels of occludin and ZO-1 were observed in LPS-treated cells, but not in cells pretreated with 100 μM FA. Compared with LPS-treated cells, 100 μM FA pretreatment increased the mRNA and protein expression of occludin and ZO-1 (*P* < 0.01). TJs ultrastructure of Caco-2 cells was investigated by transmission electron microcopy (**Figure 2F**). In the control of Caco-2 cells, TJ structure between adjoining cells was intact and densely connected. In LPS-treated Caco-2 cells, the TJ structure was obviously damaged with large gaps between adjoining Caco-2 cells and the electron-dense material was reduced. However, pretreatment with 100 μM FA significantly alleviated LPS-induced TJs disruption.

### FA increased miR-200c-3p Expression in Caco-2 Cells Stimulated With LPS

**Figure 3:**
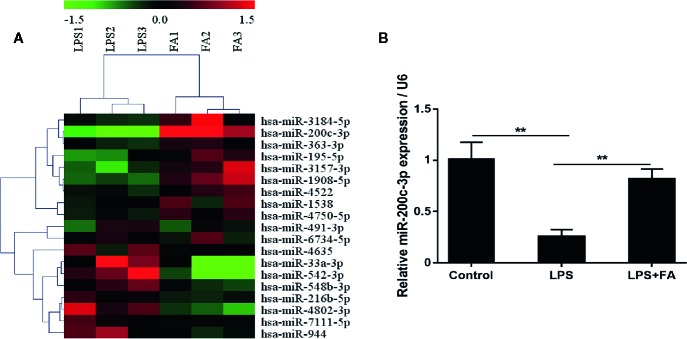
MiR-200c-3p expression was increased by FA in LPS-induced Caco-2 cells. Cells were pretreated with FA (100 μM) for 2 h and then stimulated with LPS for 24 h. **(A)** Heat map evaluation of the distinguishable miRNA expression patterns among the LPS and LPS+FA group samples. Each column represents the expression pattern of one sample, and the high expression level and the low expression level are indicated by the “red” and “green” lines, respectively. **(B)** The expression of miR-200c-3p was measured by qRT-PCR. Data were presented as means ± SD from three independent experiments and differences between means were compared using one-way ANOVA with Tukey’s multiple comparisons test. **P < 0.01.

The miRNA expression profiles of Caco-2 cells treated with LPS and FA were evaluated by microarray hybridization. Using hierarchical clustering, 19 miRNAs were found to be significantly altered in the cells pretreated with FA compared with the cells treated with LPS alone. Of these miRNAs, 10 were up-regulated and nine down-regulated (**Figure 3A**; **Table S1**). The miRNA expression profiles showed that the expression of miR-200c-3p was up-regulated 3.87-fold change in the cells pretreated with FA compared with the cells treated with LPS alone. Furthermore, we validated the expression of miR-200c-3p by qRT-PCR. The results revealed that pretreatment with FA attenuated LPS-induced reduction of miR-200c-3p expression induced by LPS in Caco-2 cells (*P* < 0.01; **Figure 3B**).

### FA Protected Caco-2 Cells Against LPS-Induced Intestinal Epithelial Barrier Dysfunction *via* miR-200c-3p

**Figure 4:**
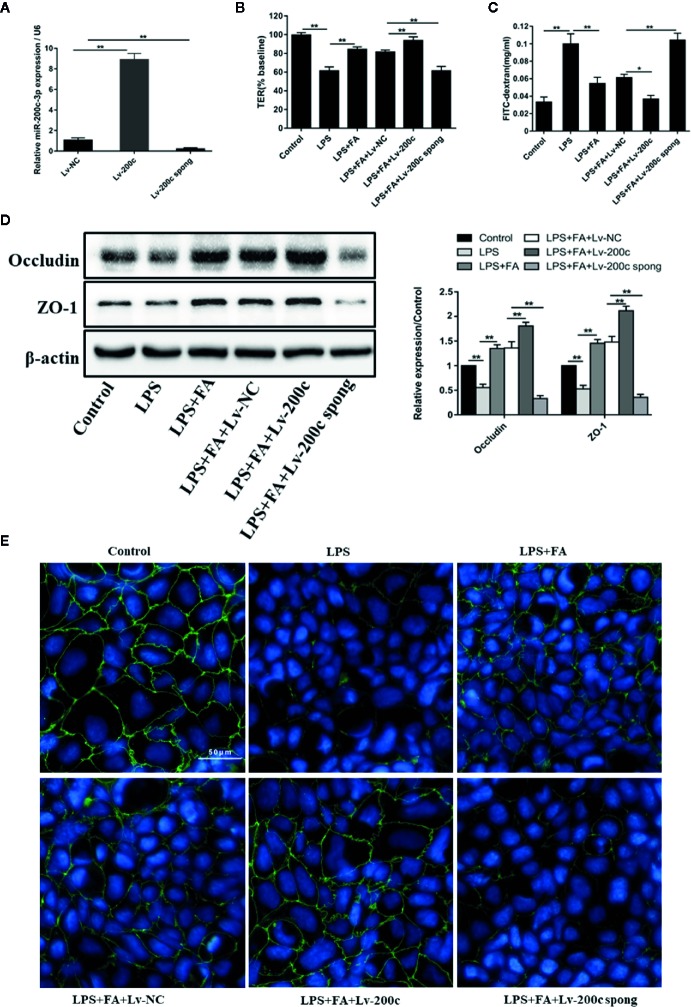
FA protected against LPS-induced intestinal epithelial barrier dysfunction by up-regulation of miR-200c-3p. **(A)** Caco-2 cells were transfected with Lv-miR-200c-3p (Lv-200c), Lv-miR-200c-3p spong (Lv-200c spong), and Lv-NC at a MOI of 15 and incubated at 37°C with 5% CO_2_ for 48 h, the expression levels of miR-200c-3p were measured by qRT-PCR. **(B, C)** Cells were transfected with Lv-200c, Lv-200c spong or Lv-NC at an MOI of 15 and incubated at 37°C with 5% CO_2_ for 48 h, and then cells were pretreated with FA (100 μM) for 2 h and stimulated with LPS for 24 h. TER values were monitored across the cell monolayers using Millicell-ERS and permeability of FD4 across the cell monolayer was measured. **(D)** The protein expression levels of occludin and ZO-1 were determined by Western blot analysis. Data were presented as means ± SD from three independent experiments and differences between means were compared using one-way ANOVA with Tukey’s multiple comparisons test. *P < 0.05, **P < 0.01. **(E)** The distribution and expression of ZO-1 were detected by immunofluorescence assay. ZO-1 (green) was labeled with fluorescent secondary antibodies and nuclei (blue) were labeled with DAPI (Scale bar = 50 μm).

The effect of miR-200c-3p or miR-491-3p on the protective effects of FA on LPS-induced intestinal epithelial barrier dysfunction was explored. Overexpression or suppression of miR-491-3p had no effect on the expression of occludin and ZO-1 (**Figure S1**). Further, **Figure 4A** showed that compared with Lv-NC transfection, the expression level of miR-200c-3p was significantly enhanced by Lv-miR-200c-3p, but was notably reduced by transfection with Lv-miR-200c-3p spong in Caco-2 cells (*P* < 0.01). Then, TER values, FD4 flux, tight junction-associated protein occludin, and ZO-1 expression were determined in transfected Caco-2 cells. The protecting effects of FA on intestinal epithelial barrier function were enhanced by transfection with Lv-miR-200c-3p, as shown by elevated TER values (*P* < 0.01, **Figure 4B**), decreased FD4 flux (*P* < 0.01, **Figure 4C**), and increased occludin and ZO-1 proteins expression (*P* < 0.01, **Figure 4D**). However, these results were reversed by suppression of miR-200c-3p (*P* < 0.05 and *P* < 0.01, **Figures 4B−D**).

Additionally, immunofluorescence staining was employed to detect the change of ZO-1 distribution in Caco-2 cells (**Figure 4E**). In the control group, ZO-1 staining appeared characteristically continuous belt-like pattern around the apical membrane of Caco-2 cells. As expected, there were striking changes in the distributions of ZO-1 in the LPS-treated cells, exhibiting irregularly undulating and staining intensity decreasing. In Caco-2 cells pretreated with FA, the rearrangements of ZO-1 were markedly attenuated, presenting a relatively unambiguous profile. Moreover, miR-200c-3p overexpression further improved the distributions of ZO-1. However, suppression of miR-200c-3p weakened the protective effects of FA on LPS-induced ZO-1 distributions.

### FA Attenuated LPS-Induced Intestinal Epithelial Barrier Dysfunction *via* Activating microRNA-200c-3p-Mediated PI3K/AKT Pathway

**Figure 5:**
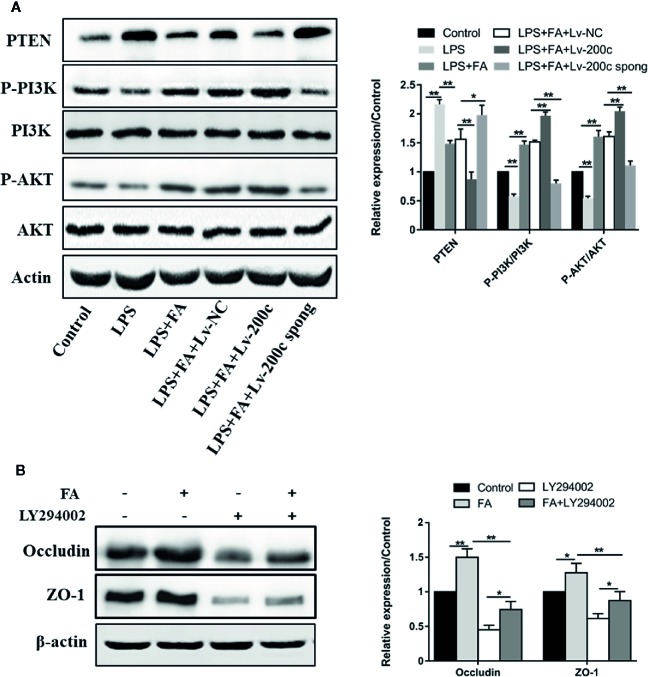
FA attenuated LPS-induced intestinal epithelial barrier dysfunction *via* activating miR-200c-3p-mediated PTEN/AKT pathway in Caco-2 cells. **(A)** Cells were transfected with Lv-200c, Lv-200c spong and Lv-NC at an MOI of 15 and incubated at 37°C with 5% CO_2_ for 48 h, and then cells were pretreated with FA (100 μM) for 2 h and stimulated with LPS for 24 h. The expression levels of PTEN, PI3K, p-PI3K, AKT and p-AKT were evaluated using western blotting. **(B)** Caco-2 cells were pretreated with 10 μM LY294002 for 1 h and then treated with 100 μM FA for 2 h. The expression levels of occludin and ZO-1 proteins were evaluated using Western blotting. Data were presented as means ± SD from three independent experiments and differences between means were compared using one-way ANOVA with Tukey’s multiple comparisons test. *P < 0.05; **P < 0.01.

To clarify the mechanism underlying FA protecting Caco-2 cells against LPS-induced intestinal epithelial barrier dysfunction, the PTEN/PI3K/AKT pathway was examined by western blotting. As shown in **Figure 5A**, LPS treatment increased PTEN protein expression (*P* < 0.01) and decreased expression of phosphorylated PI3K and AKT (*P* < 0.01). However, compared with LPS-treated cells, cells pretreated with FA had lower expression of PTEN and higher expression of phosphorylated PI3K and AKT (*P* < 0.01). FA alone increased the expression of phosphorylated PI3K and AKT (**Figure S2**). Moreover, the overexpression of miR-200c-3p significantly strengthened the effect of FA on the PTEN/PI3K/AKT pathway (*P* < 0.01). However, suppression of miR-200c-3p notably inhibited the effect of FA on PTEN/PI3K/AKT pathway in LPS-treated cells (*P* < 0.05 or *P* < 0.01).

Subsequently, LY294002, the inhibitors of PI3K/Akt signaling pathway was used to suppress PI3K/Akt activity. The expressions of occludin and ZO-1 proteins were then examined. Compared with control cells, increased expression of occludin and ZO-1 proteins were observed in Caco-2 cells pretreated with FA alone, but not in cells treated with LY294002 alone (*P* < 0.01, **Figure 5B**). FA-induced increase in the expression of occludin and ZO-1 proteins was prevented by LY294002 (*P* < 0.01).

## Discussion

Our previous studies using the rat IEC-6 cell model demonstrated that FA protects against heat stress-induced intestinal epithelial barrier dysfunction *via* the PI3K/AKT-mediated Nrf2/HO-1 signaling pathway (He et al., 2016; He et al., 2018). In humans, dysfunction of intestinal epithelial barrier leads to increased permeability of intestinal mucosa, subsequent translocation of intestinal pathogenic bacteria or toxins, which in turn aggravates the damage of intestinal barrier integrity, resulting in local intestinal or systemic disease such as inflammatory bowel diseases, multiple organ dysfunction syndromes, and sepsis (Yoseph et al., 2016; Meng et al., 2017). LPS, a representative biological marker of systemic microbial translocation, plays an important role in the initiation and development of intestinal epithelial barrier dysfunction (Nighot et al., 2017). Caco-2 cells infected with LPS are widely used as an *in vitro* model for evaluating intestinal barrier function in humans. Thus, in the present study, we aimed to investigate whether FA attenuates LPS-induced barrier dysfunction through PI3K/AKT signaling in Caco-2 cells. The results demonstrated that FA pretreatment inhibited LPS-induced increase in intestinal permeability and restored tight junction structure integrity in Caco-2 cells. Moreover, FA enhanced the expression of miR-200c-3p in Caco-2 cells treated with LPS, which negatively regulates the PTEN activity and thus promoted the downstream activation of PI3K/AKT pathway. Our study provided the first clarification into the potential mechanism underlying FA protection against LPS-induced intestinal epithelial barrier dysfunction *via* microRNA-200c-3p-mediated activation of PI3K/AKT signaling pathway.

We found that LPS strikingly disrupted intestinal barrier function of Caco-2 cells, accompanied by decreased TER values, elevated FD4 flux, and decreased occludin and ZO-1 protein expression. It was reported that FA competitively binds to monocarboxylic acid transporter to inhibit the transepithelial transport of fluorescein in Caco-2 cells (Konishi et al., 2002). In LPS-stimulated RAW 264.7 cells, 100 μM FA reduces the translocation of NF-E2-related factor 2 (Nrf2) and nuclear transcription factor-κB (NF-κB) into the nuclei through a reduction of the expression of phosphorylated IKK and consequently inhibits proinflammatory IL-6 production (Lampiasi and Montana, 2016; Lampiasi and Montana, 2018). FA down-regulates mitogen-activated protein kinase signaling (p38, ERK and JNK) and reserved the antioxidant activity in the injured lungs induced by LPS stimulation (Zhang et al., 2018). Several studies also demonstrated that FA may target Toll-like receptor 4 (TLR4) to inhibit LPS infection. FA has been shown potentially effective therapeutic agent in the acute kidney injury model of LPS stimulation through suppression of inflammatory events by inhibiting TLR-4 mediated NF-κB activation (Mir et al., 2018). FA suppresses LPS-induced TLR4 activation to reduce the expression of proinflammatory tumor necrosis factor α and interleukin 1β in macrophages (Navarrete et al., 2015). FA interferes with the TLR4/MD2 complex binding site and rescues LPS-induced neurotoxicity in the mouse hippocampus (Rehman et al., 2019). However, the effect of FA on LPS-induced intestinal barrier dysfunction remains unclear. In this study, we found that FA pretreatment reversed the LPS-induced intestinal epithelial barrier dysfunction by decreasing epithelial permeability, increasing the expression of occludin and ZO-1 proteins, and maintaining tight junction integrity. These results indicated that FA has a protective potential to prevent LPS-induced intestinal epithelial barrier dysfunction.

The important role of miRNAs in regulating intestinal epithelial barrier function has been well established (Ye et al., 2011). In the present study, the analysis of miRNA expression sequencing showed several significantly different miRNAs, including miR-491-3p (fold change: 16.32), miR-4635 (fold change: 10.33), miR-1538 (fold change: –10.11), miR-6734-5p (fold change: –5.36), miR-7111-5p (fold change: –4.90), miR-4750-5p (fold change: 4.21), and miR-200c-3p (fold change: 3.87). The function and molecular mechanism of miR-4635, miR-1538, miR-6734-5p, miR-7111-5p, or miR-4750-5p have not been reported. Studies suggested that miR-491-3p may be involved in attenuating multidrug resistance of hepatocellular carcinoma, or suppressing the growth and invasion of hepatocellular carcinoma (Rodrigues et al., 2016; Duan et al., 2017; Zhao et al., 2017). Indeed, we also found that overexpression or suppression of miR-491-3p had no effect on the expression of occludin and ZO-1. Emerging studies have found that the miR-200c-3p has an important role in multiple diseases, including ovarian cancer, breast cancer, and gastric cancer (Sulaiman et al., 2016; Ghasabi et al., 2019). The miR-200 family includes miR-200a, miR-200b, miR-200c, miR-141, and miR-429. Among them, it has been reported that miR-429 can down-regulate the expression of Ocln by targeting the 3′-UTR of Ocln mRNA, leading to the destruction of tight junction and enhanced intestinal barrier permeability (Yu et al., 2016). In addition, miR-200b has been demonstrated to attenuate tight junction injury induced by tumor necrosis factor α through targeting c-Jun and MLCK (Shen et al., 2017). We found that FA significantly inhibited the decreased expression of miR-200c-3p induced by LPS. Furthermore, overexpressed miR-200c-3p remarkably enhanced the protective effect of FA on LPS-induced intestinal barrier dysfunction involving in increasing cell permeability and maintaining tight junction protein functions, however, knockdown of miR-200c-3p reversed the protective effect of FA on LPS-induced intestinal barrier dysfunction. These findings indicated that FA protected Caco-2 cells from LPS-induced intestinal barrier dysfunction at least in part by up-regulating the expression of miR-200c-3p. Further study is needed to explore the mechanism of FA in improving intestinal diseases *via* regulating other miRNAs with high fold changes.

MiR-200c-3p regulates multiple biological processes by binding to different target coding genes. PTEN gene has been reported as a target of miR-200c-3p. PTEN dephosphorylates phosphatidylinositol−3,4,5−trisphosphate (PIP3), which recruits AKT to the cell membrane and is phosphorylated by other kinases dependent on PIP3 and PTEN negative regulates the PI3K/AKT signaling pathway (Tokuhira et al., 2015; Hu et al., 2019). Recent literature has shown that PI3K/AKT signaling pathway contributes to the regulation of tight junctions. Resveratrol has been reported to attenuate oxidative stress-induced intestinal barrier injury *via* PI3K/AKT-mediated Nrf2 signaling pathway (Zhuang et al., 2019). PI3K/AKT/mTOR pathway is involved in the regulation of the blood-brain barrier’s tight junction proteins during arsenic-induced autophagy in the developmental mouse cerebral cortex and hippocampus (Manthari et al., 2018). Activation of the PI3K/AKT pathway as a result of TLR2 activation has also been shown to enhance epithelial barrier integrity. TLR2 augments the intestinal barrier through ZO-1 protein redistribution in response to stress-induced damage under control of the PI3K/AKT pathway (Cario et al., 2007). Several studies suggested that FA inhibits proliferation and induces apoptosis *via* down-regulating PI3K/AKT signaling pathway dose-dependently and exerts anti-cancer properties in some cancer cells, including 143B and MG63 osteosarcoma cells (30, 100, and 150 μM treatment) (Wang et al., 2016) and Caski human cervical carcinoma cells (4–20 μM treatment) (Luo et al., 2020). FA treatment (10 – 30 μM) reverses P-glycoprotein mediated multidrug resistance *via* inhibition of PI3K/AKT/NF-κB signaling pathway in KB Ch^R^8-5 cells (Muthusamy et al., 2019). A caffeic acid-ferulic acid hybrid compound attenuates LPS-mediated inflammatory responses partly through suppressing PI3K/AKT/NF-κB signaling pathway in murine BV2 and RAW264.7 cells (Kwon et al., 2019). It was also reported that 5 μM FA protects human umbilical vein endothelial cells from radiation-induced oxidative stress *via* activating PI3K/AKT signaling pathway (Ma et al., 2010). Our previous study found that FA has a protective effect on intestinal epithelial barrier function in IEC-6 cells suffering from heat stress, which is associated with activation of the antioxidant PI3K/AKT/Nrf2/HO-1 signaling pathway (He et al., 2016; He et al., 2018). In the present study, FA increased the expression of phosphorylated PI3K and AKT. Difference of use dose and cell species may contribute to various physiological function of FA and distinct regulatory effects of FA on PI3K/AKT signaling pathway. Previous research has demonstrated that miR-21 can regulate intestinal epithelium tight junction permeability involving in PTEN/PI3K/AKT signaling pathway in Caco-2 cells (Zhang et al., 2015). Thus, miR-200c-3p might be participated in the regulation of intestinal barrier function by regulating the PTEN/PI3K/AKT signaling pathway. In the present study, we investigated whether FA affected the PTEN/PI3K/AKT signaling pathway to improve the intestinal barrier function through regulating miR-200c-3p expression. Results showed that FA significantly inhibited PTEN activity by LPS and promoted activation of PI3K/AKT signaling pathway. Moreover, FA-mediated activation of PI3K/AKT pathway was notably strengthened by miR-200c-3p overexpression and inhibited by miR-200c-3p suppression. These findings suggested that FA up-regulates miR-200c-3p and then negatively regulatory promotes activation of PTEN/PI3K/AKT pathway, thereby protecting against LPS-induced Caco-2 cell barrier dysfunction.

**Figure 6:**
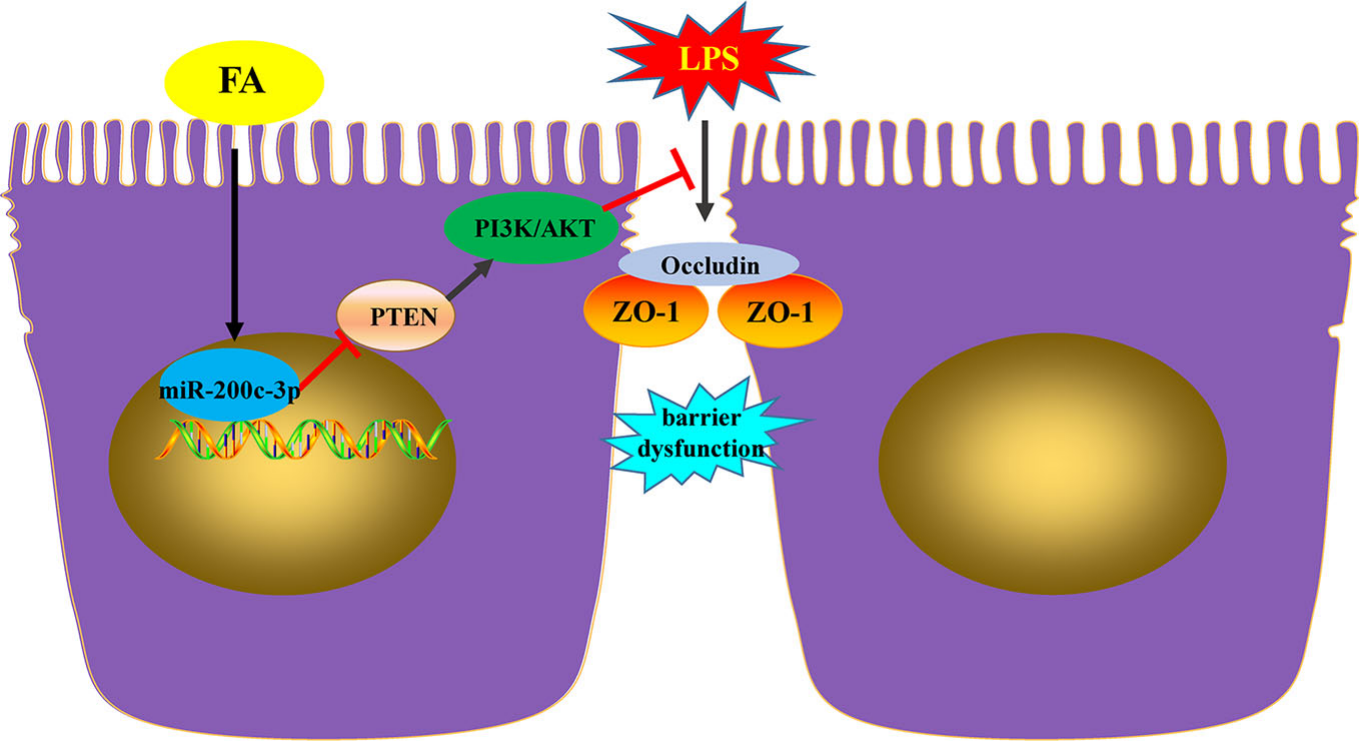
FA ameliorated lipopolysaccharide-induced barrier dysfunction *via* miR-200c-3p-mediated activation of PI3K/AKT pathway in Caco-2 cells. FA exhibited the protective effects on LPS-induced barrier dysfunction involving in miR-200c-3p and PTEN/PI3K/Akt signaling pathway. FA promotes activation of PI3K/AKT pathway by miR-200c-3p-mediated suppression of the negative mediator PTEN, which, in turn, maintains TJ function and thus ameliorates LPS-induced intestinal epithelial barrier dysfunction.

In conclusion, the present study demonstrated that in Caco-2 cells, FA exhibited the protective effects on LPS-induced barrier dysfunction involving in miR-200c-3p and PTEN/PI3K/Akt signaling pathway. FA promotes activation of PI3K/AKT pathway by miR-200c-3p-mediated suppression of the negative mediator PTEN, which, in turn, maintains TJ function and thus ameliorates LPS-induced intestinal epithelial barrier dysfunction (**Figure 6**). These findings provide new insights for interpreting the potential molecular mechanisms of FA-mediated protective effects on intestinal epithelial barrier function. FA may be a candidate drug for the treatment of intestinal barrier dysfunction-related diseases. More animal or clinical experiments are essential to further evaluate the protective effect of FA on intestinal epithelial barrier dysfunction.

## Data Availability Statement

The microarray data generated for this study has been deposited at Sequence Read Archive under the accession number PRJNA588301.

## Author Contributions 

SH, QL, and YG conceived and designed the experiments. SH, XX, JZ, and NW performed the experiments. SH, and XX analyzed the data. NW contributed reagents/materials/analysis tools. SH wrote the paper.

## Funding 

This work was supported by grants from the National Natural Science Foundation of China (Nos. 81803879, 81673934, and 81973608), Beijing Natural Science Foundation (7192083), and the National Major Scientific and Technological Project (No. 2017ZX10305501).

## Conflict of Interest

The authors declare that the research was conducted in the absence of any commercial or financial relationships that could be construed as a potential conflict of interest.

## Supplementary Material

Table S1 List of differentially expressed miRNAs in Caco2 cells of FA pretreatment compared with the LPS treatment without FA.

Figure S1 The effect of miR-491-3p in FA protection against LPS-induced intestinal epithelial barrier dysfunction. Caco-2 cells were transfected with Lv-miR-491-3p and Lv-miR-491-3p spong at an MOI of 20 and incubated for 48 h at 37°C with 5% CO2. The protein expression levels of occludin and ZO-1 were determined by Western blot analysis.

Figure S2 FA activated PI3K/AKT pathway in Caco-2 cells. Caco-2 cells were treated with 100 μM FA for 2 h. The expression levels of PI3K, p-PI3K, AKT, and p-AKT were evaluated using Western blotting. Data were presented as means ± SD from three independent experiments and differences between means were compared using the unpaired t test. **P< 0.01.
