# Supplementary material for: Ferulic Acid Ameliorates Lipopolysaccharide-Induced Barrier Dysfunction via MicroRNA-200c-3p-Mediated Activation of PI3K/AKT Pathway in Caco-2 Cells
Source: Front Pharmacol. 2020 Apr 3;11:376. doi: 10.3389/fphar.2020.00376 (PMC7145943; doi:10.3389/fphar.2020.00376)
Supplement: Table S1 — List of differentially expressed miRNAs in Caco2 cells of FA pretreatment compared with the LPS treatment without FA. [file Table_1.docx]

**Table S1. List of differentially expressed miRNAs in Caco2 cells of FA pretreatment compared with the LPS treatment without FA.**

| **miRNA** | | **FA/LPS Fold change**  **(Ratio ≥ 1.5)** | | **P-value** | |
| --- | --- | --- | --- | --- | --- |
| hsa-miR-1538 | -10.11400 | 0.03710 | |  |  |
| hsa-miR-6734-5p | -5.35896 | 0.03995 | |  |  |
| hsa-miR-7111-5p | -4.90061 | 0.04405 | |  |  |
| hsa-miR-548b-3p | -3.12679 | 0.00936 | |  |  |
| hsa-miR-4802-3p | -2.38456 | 0.00025 | |  |  |
| hsa-miR-216b-5p | -2.32365 | 0.04348 | |  |  |
| hsa-miR-944 | -1.83248 | 0.03272 | |  |  |
| hsa-miR-542-3p | -1.55814 | 0.00009 | |  |  |
| hsa-miR-33a-3p | -1.52233 | 0.00070 | |  |  |
| hsa-miR-1908-5p | 1.51352 | 0.01667 | |  |  |
| hsa-miR-3157-3p | 1.69286 | 0.00773 | |  |  |
| hsa-miR-195-5p | 1.83772 | 0.04456 | |  |  |
| hsa-miR-3184-5p | 2.16612 | 0.01360 | |  |  |
| hsa-miR-4522 | 2.61339 | 0.03206 | |  |  |
| hsa-miR-363-3p | 3.28197 | 0.03125 | |  |  |
| hsa-miR-200c-3p | 3.87435 | 0.00037 | |  |  |
| hsa-miR-4635 | 10.32715 | 0.03794 | |  |  |
| hsa-miR-4750-5p | 4.20970 | 0.02275 | |  |  |
| hsa-miR-491-3p | 16.32553 | 0.00645 | |  |  |
|  | | | | | |
