# Supplementary figures and images for: Ferulic Acid Ameliorates Lipopolysaccharide-Induced Barrier Dysfunction via MicroRNA-200c-3p-Mediated Activation of PI3K/AKT Pathway in Caco-2 Cells
Source: Front Pharmacol. 2020 Apr 3;11:376. doi: 10.3389/fphar.2020.00376 (PMC7145943; doi:10.3389/fphar.2020.00376)

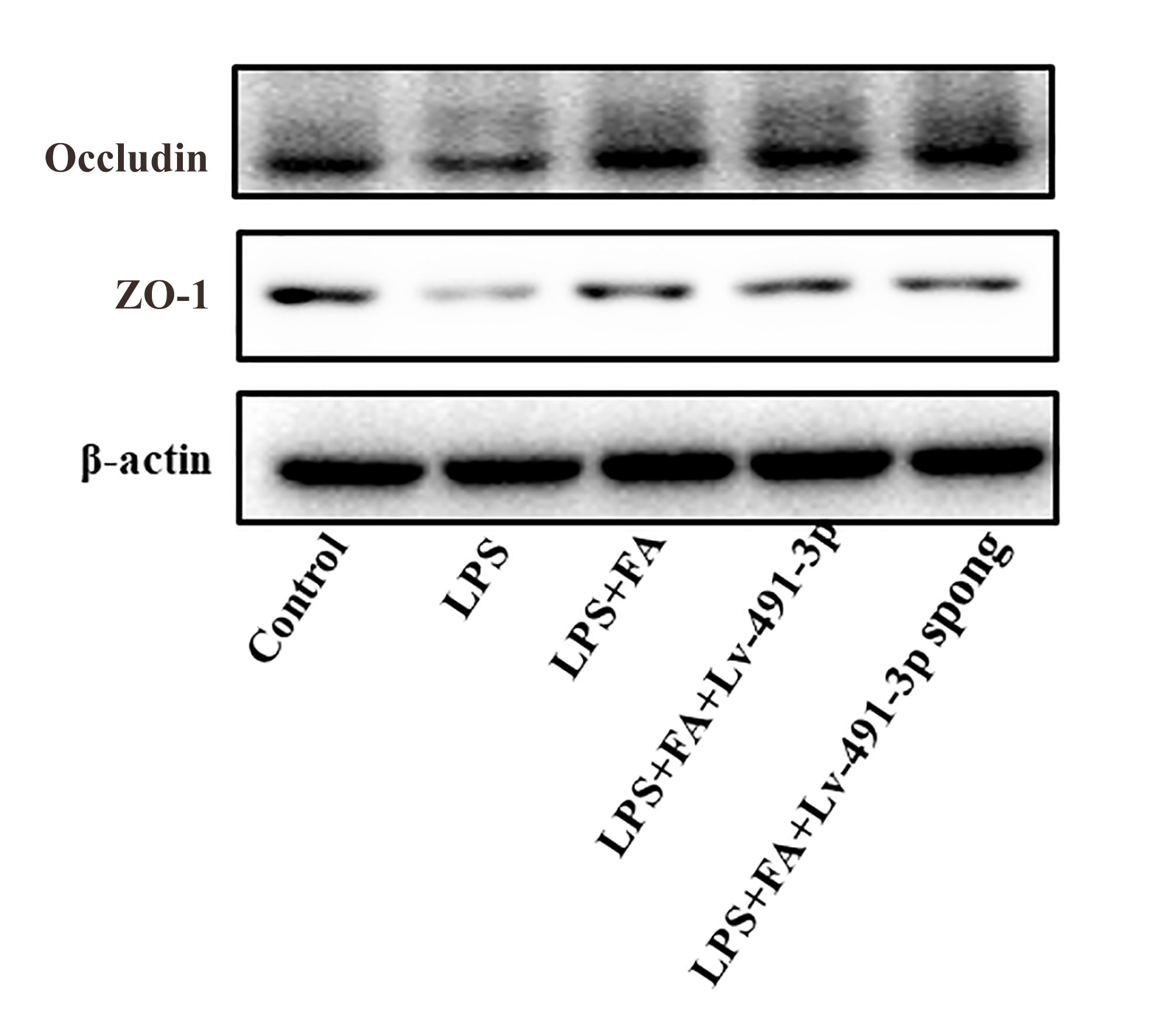

Supplement: Figure S1 — The effect of miR-491-3p in FA protection against LPS-induced intestinal epithelial barrier dysfunction. Caco-2 cells were transfected with Lv-miR-491-3p and Lv-miR-491-3p spong at an MOI of 20 and incubated for 48 h at 37°C with 5% CO2. The protein expression levels of occludin and ZO-1 were determined by Western blot analysis. [file Image_1.tif]

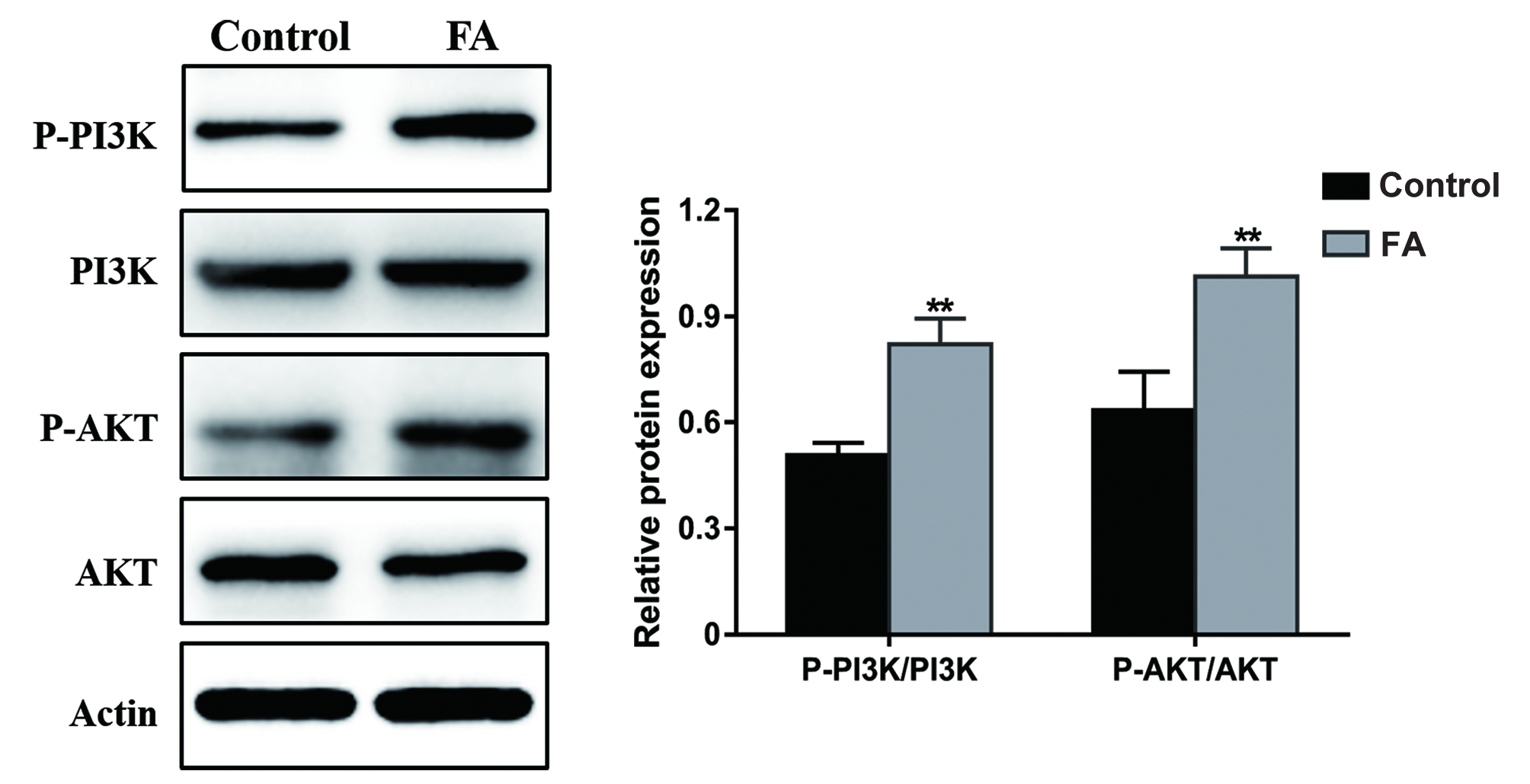

Supplement: Figure S2 — FA activated PI3K/AKT pathway in Caco-2 cells. Caco-2 cells were treated with 100 μM FA for 2 h. The expression levels of PI3K, p-PI3K, AKT, and p-AKT were evaluated using Western blotting. Data were presented as means ± SD from three independent experiments and differences between means were compared using the unpaired t test. **P< 0.01. [file Image_2.tif]
